# ROS induced distribution of mitochondria to filopodia by Myo19 depends on a class specific tryptophan in the motor domain

**DOI:** 10.1038/s41598-017-11002-9

**Published:** 2017-09-14

**Authors:** Boris I. Shneyer, Marko Ušaj, Naama Wiesel-Motiuk, Ronit Regev, Arnon Henn

**Affiliations:** Faculty of Biology, Technion - Israel Institute of Technology, Haifa, 3200003 Israel

## Abstract

The role of the actin cytoskeleton in relation to mitochondria function and dynamics is only recently beginning to be recognized. Myo19 is an actin-based motor that is bound to the outer mitochondrial membrane and promotes the localization of mitochondria to filopodia in response to glucose starvation. However, how glucose starvation induces mitochondria localization to filopodia, what are the dynamics of this process and which enzymatic adaptation allows the translocation of mitochondria to filopodia are not known. Here we show that reactive oxygen species (ROS) mimic and mediate the glucose starvation induced phenotype. In addition, time-lapse fluorescent microscopy reveals that ROS-induced Myo19 motility is a highly dynamic process which is coupled to filopodia elongation and retraction. Interestingly, Myo19 motility is inhibited by back-to-consensus-mutation of a unique residue of class XIX myosins in the motor domain. Kinetic analysis of the purified mutant Myo19 motor domain reveals that the duty ratio (time spent strongly bound to actin) is highly compromised in comparison to that of the WT motor domain, indicating that Myo19 unique motor properties are necessary to propel mitochondria to filopodia tips. In summary, our study demonstrates the contribution of actin-based motility to the mitochondrial localization to filopodia by specific cellular cues.

## Introduction

Mitochondria participate in multiple cellular functions including ATP synthesis, calcium buffering, lipid synthesis, signaling and apoptosis^1^. The distribution of mitochondria throughout the cell is regulated and affected by external stimuli such as stress or growth factors^2, 3^. Consistent with the critical roles of the mitochondria, aberrant distribution of these organelles is implicated in neurodegenerative diseases^4–6^. Mitochondria move primarily on microtubule (MT) tracks via kinesin and dynein motors, although some motility is mediated via the actin cytoskeleton^7–9^.

Myosins are actin-based motors that participate in diverse cellular functions including muscle contractions, cytoskeleton dynamics, membrane tension and cargo transport^10, 11^. Their involvement in human diseases such as cancer is coming to be appreciated at the molecular level^12, 13^. The myosin architecture contains a highly conserved motor domain that binds actin and ATP, followed by a ‘neck domain’ that possesses the light–chain-binding motifs, and a highly variable C-terminal domain that interacts with diverse cargo, proteins and membranes^14^. Despite the high degree of conservation of the motor structure and the conserved ATPase cycle across all myosin classes and isoforms, the rate and equilibrium constants of the biochemical transitions vary greatly, yielding a unique enzymatic adaptation tailored for each individual myosin’s cellular function^15–17^.

Myosin 19 (Myo19) is a mitochondrial actin-based molecular motor involved in mitochondrial motility, segregation during cell division, and localization to filopodia tips^18–20^. Myo19 is embedded in the outer mitochondrial membrane via a highly specific and stable interaction mediated by a unique ~45 amino acids motif in its C-terminal domain^20, 21^.

Filopodia, thin actin-based protrusions in the cell membrane that allow the cell to probe the environment, function in a variety of cellular activities such as cell motility, wound healing, and phagocytosis, and are also precursors of dendritic spines^22, 23^. Filopodia form by linear polymerization of G-actin-ATP at their barbed–ends mediated by formins at the tip, a process regulated by small Rho family GTPases and promoted by oxidative stress and reactive oxygen species (ROS)^23–25^. ROS exert deleterious effects on the cell by damaging lipids, proteins and DNA^26^. However, a physiological level of oxidative stress is important for normal cellular functions, and ROS also serve as secondary messengers in signaling cascades that affect actin cytoskeleton rearrangement, proliferation, differentiation, cell motility and apoptosis. ROS affect signaling pathways by oxidizing reactive cysteins found in the active site of phosphatases and kinases, thereby inhibiting phosphatases and modulating kinases activities^26, 27^.

We extended our studies of the effect of glucose starvation on Myo19, and found that the localization of Myo19 and mitochondria to filopodia tips is mediated by ROS. Tracking Myo19 localization during ROS-induced filopodia formation, revealed its inducible motor activity that strongly supports active and directional motility of Myo19-bound mitochondria towards filopodia tips. A highly conserved amino acid in the motor domain at the end of P-loop and beginning of Loop 1 (position 140) is unique to class XIX^28^. Mutation of this position to the consensus amino acid of *Human* myosins (W140V) affected its motility to the filopodia tips, although its localization to the mitochondria remained unchanged. To study the protein’s motor properties, we co-expressed and purified the mutant Myo19^W140V^-3IQ with calmodulin as a light chain in a suspension adapted, serum-free, human HEK293SF-3F6 cell line. Steady-state and transient kinetics, as well as kinetic modeling, allowed us to identify key features that differed from the WT and were affected by the mutation. Strikingly, gentle modulation of Myo19 ATPase cycle parameters strongly affected its cellular function as revealed by the Myo19^W140V^ mutant phenotype.

## Results

### Glucose starvation–induced Myo19 localization is mediated by ROS

Glucose starvation increases the cellular levels of ROS by increasing ROS generation by both mitochondria and NADPH oxidase (NOX) enzymes^29^. NOX enzymes reside on the plasma membrane and oxidize extracellular oxygen to generate ROS that enter the cell^30, 31^. The resultant increase in oxidative stress inhibits phosphatases and induces a specific phospho-tyrosine phosphorylation signature associated with focal adhesions^29^.

**Figure 1:**
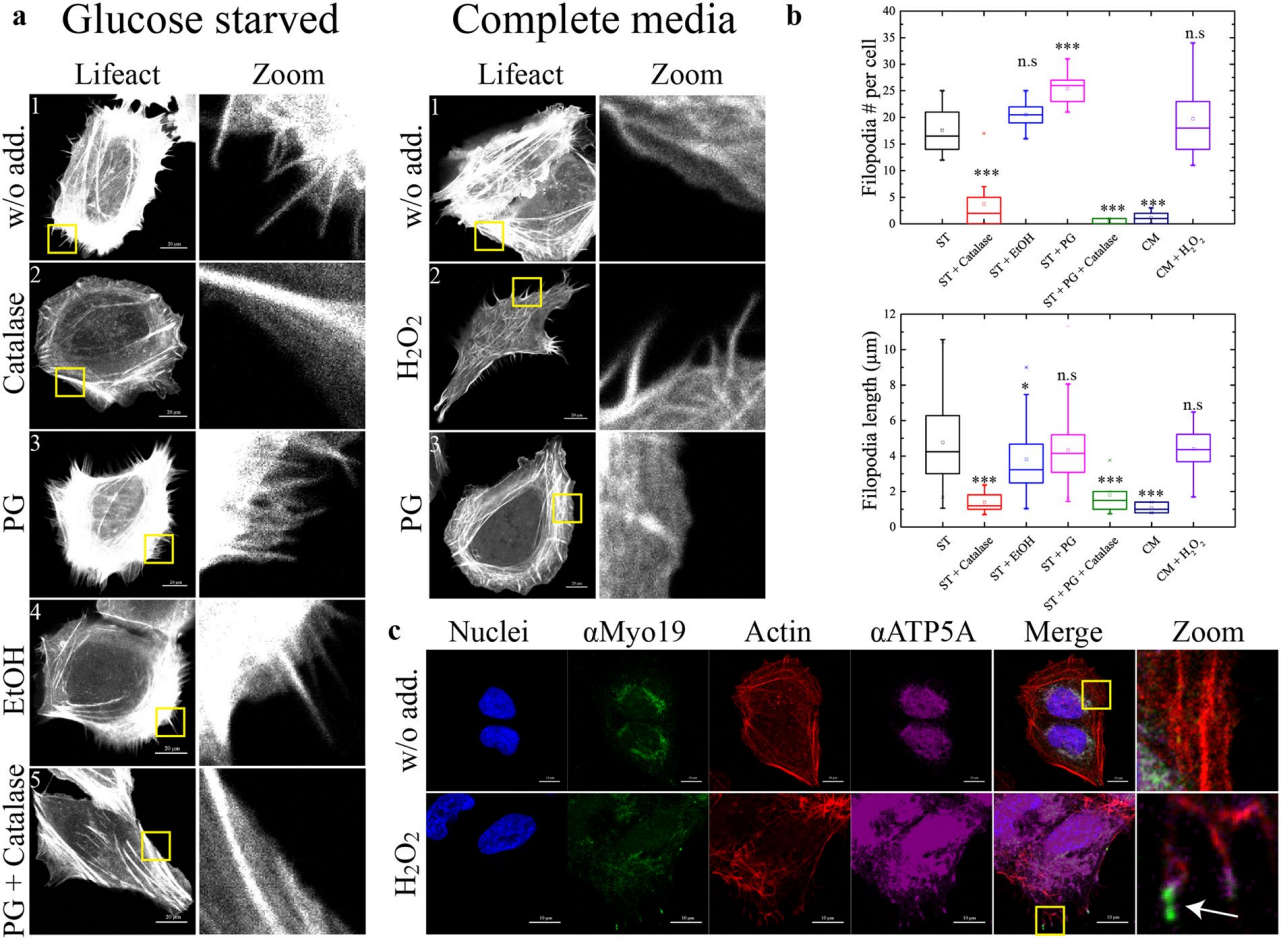
Glucose-starvation induction of filopodia formation is mediated by ROS. (**a**) Left panel: Filopodia induced by glucose-starvation are inhibited by supplementation of antioxidants to the starvation media. emLifeact (fluorescently tagged actin binding protein) was transiently expressed in U2OS cells to visualize actin and filopodia. The cells were then starved in starvation medium without supplements or supplemented with either catalase (60 µg/ml), Propyl Gallate (PG, 20 µM) or a combination of both (EtOH was used as vehicle). Right panel: ROS induces filopodia formation. emLifeact expressing U2OS cells were exposed to either PG or 0.2 mM H_2_O_2_ for two hrs, inducing filopodia formation in complete growth media. Note the lack of effect of PG in complete medium compared to starvation medium. Bar is 20 µm. (**b**) Quantification of the number and length of the filopodia in (**a**). Over 300 filopodia were manually counted for each condition from three independent experiments. The significance of the change in both number and length of filopodia was assessed via two tailed Student’s T-test. pVal for filopodia number per cell = 1.4 × 10^−6^, 0.083, 0.0001, 3.91 × 10^−11^, 3.57 × 10^−11^, 0.4.pVal for filopodia length = 1.27 × 10^−14^, 0.01, 0.093, 0.0027, 0.0035, 0.161 (same order as the chart). (**c**) Immunofluorescence for endogenous Myo19 in H_2_O_2_ stimulated U2OS cells reveals that it localizes with mitochondria at filopodia tips. Blue – nuclei, green – αMyo19, red – phalloidin stained actin, Magenta – The mitochondrial marker ATP5A (ATP-synthase subunit alpha). Bar is 10 µm. Zoom – magnification of the area indicated by the yellow rectangle. We note that the staining of ATP5A is also nuclear, however this is due to the secondary antibody used for detection of ATP5A stains which stains nuclei even in the absence of the ATP5A antibody.

We sought to investigate the connection between the effects of glucose starvation (and elevated ROS) and Myo19 activation, as well as determine whether the starvation-induced localization of Myo19 is indeed mediated by ROS. To visualize filopodia under starvation conditions with or without the addition of antioxidants, we transfected U2OS cells with the fluorescently labeled actin binding protein Emerald-LifeAct (emLifeAct) (Fig. 1a). Consistent with results obtained previously using fascin as a filopodia marker, starvation of the emLifeAct-expressing cells resulted in filopodia formation (17.6 ± 3.9 filopodia per cell, 4.77 ± 2.1 µm in length, N = 100 from three independent experiments, Fig. 1a,b). These cells formed fewer filopodia than cells overexpressing fascin, likely due to the bundling activity resulting from fascin overexpression^20, 32^. Interestingly, addition of the extracellular H_2_O_2_ scavenger catalase to the starvation media strongly inhibited filopodia formation, indicating that the signal for filopodia formation is mediated by extracellular H_2_O_2_ (Fig. 1a, left panel: 2, Fig. 1b). The addition of the common antioxidant propyl gallate (PG) to starvation media had a synergistic effect on filopodia formation, increasing their number but not their length, but had no effect when added to non-starved cells (Fig. 1a, right panel: 3, Fig. 1b). This surprising and counterintuitive finding can be explained by the fact that PG is a dismutase mimic and can increase the rate at which extracellular non-permeable ROS such as superoxide is converted to H_2_O_2_, enters the cell, and integrates into signaling pathways^33^. Consistent with this, the synergistic effect was completely inhibited by supplementation with catalase (Fig. 1a, left panel: 5, Fig. 1b). Taken together, these results support a mechanism in which glucose starvation induces an increase in cellular oxidative stress, which in turn induces filopodia formation. This prompted us to check whether direct addition of H_2_O_2_ to the media was sufficient to induce filopodia. Remarkably, H_2_O_2_ was able to mimic the glucose starvation–induced formation of filopodia (Fig. 1a, right panel: 2, Fig. 1b), which contained mitochondria (detected via antibody against ATP-synthase subunit alpha – ATP5A) and endogenous Myo19 (magenta and green, respectively, marked with arrow) at their tips (Fig. 1c).

### Myo19 localization to filopodia is selective

**Figure 2:**
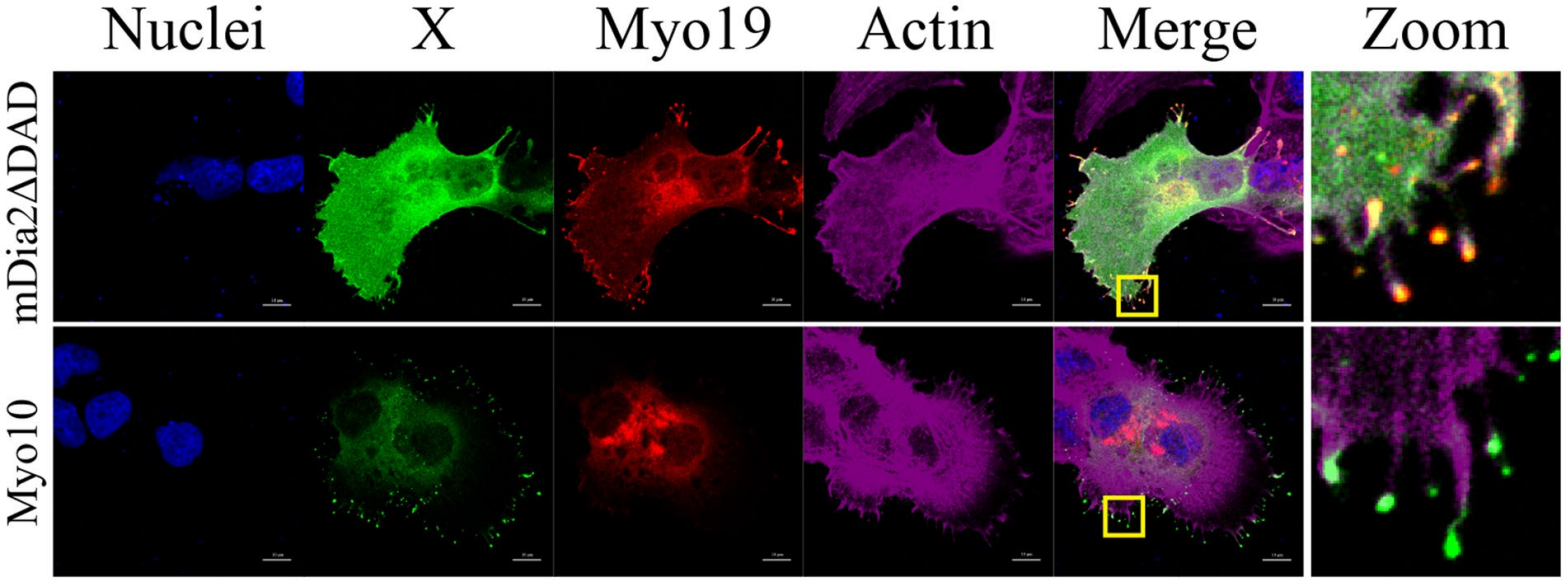
Myo19 selectively localizes to a subset of filopodia. U2OS cells were co-transfected with ruby tagged Myo19 and either GFP tagged Myo10 or GFP tagged mDia2ΔDAD (constitutively active form of the formin mDia2) that induce filopodia formation. Both Myo10 and mDia2ΔDAD expressing cells exhibit multiple filopodia, however Myo19 localized only to mDia2ΔDAD induced filopodia tips. Blue – nuclei, green – Myo10 or mDia2ΔDAD, red – ruby tagged Myo19, Magenta – phalloidin stained actin. Bar is 10 µm.

Myo19 is a plus end–directed myosin that slides on actin filaments towards their barbed ends, which are located at filopodia tips^23, 34^. This property suggests that, without regulation of its motility, Myo19 would reach the tips of all actin protrusions. To determine whether Myo19 localization to filopodia is regulated, we investigated whether Myo19 localizes to tips of filopodia induced by expression of either a constitutively active form of the formin mDia2, mDia2ΔDAD, or Myo10^35, 36^. We co-transfected U2OS cells with Ruby-tagged Myo19 and GFP-tagged forms of mDia2ΔDAD or Myo10, and followed the localization of Myo19 in these cells. Both mDia2ΔDAD- and Myo10-expressing cells spontaneously formed filopodia in complete media; however, Myo19 localized to filopodia tips only in cells expressing mDia2ΔDAD, but not in those expressing Myo10 (Fig. 2). The selective localization of Myo19 to mDia2ΔDAD-induced filopodia is likely due to differences in the modes of filopodia induction. Dimerization of Myo10 motor domains is sufficient to promote filopodia formation, relying on the bundling activity of the two motor heads^37^. By contrast, mDia2 interacts with numerous proteins, such as the tyrosine kinase Src, that may initiate a signaling cascade resulting in activation of Myo19^38^. These results suggest that Myo19 localization to filopodia tips is not the default preference, and that Myo19 localization is limited to certain actin-based protrusions.

### Myo19 tracks filopodia tip growth

**Figure 3:**
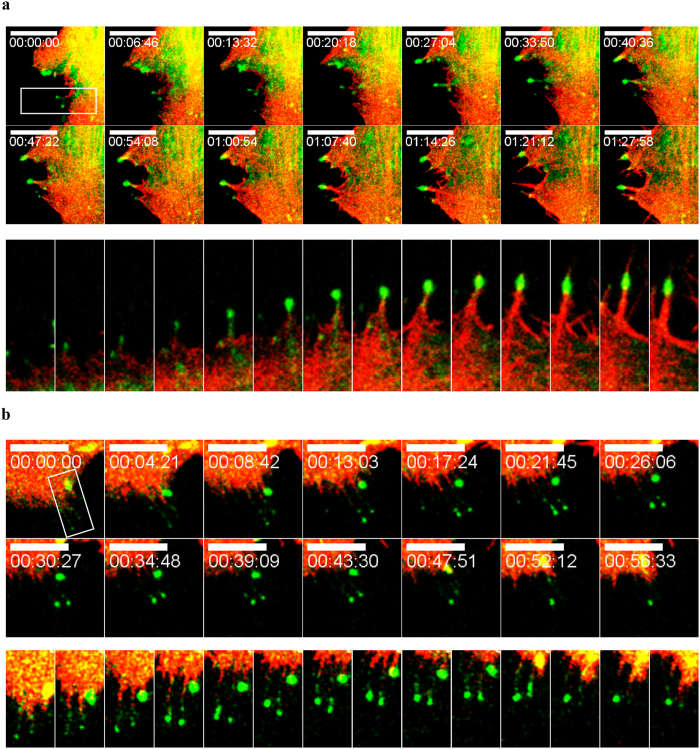
Dynamics of emMyo19 localization to filopodia tips. Montage of fluorescent microscopy images from a time-lapse movie revealing the dynamic nature of emMyo19 motility and localization to filopodia tips and back to the cell body following 0.2 mM H_2_O_2_ stimulation. (**a**) Anterograde motility of emMyo19 towards filopodia tips (**b**) Retrograde motility of emMyo19 towards the cell body. Kymographs of the boxed region are presented under each panel. Green – emMyo19, Red- Ruby-Lifeact. Bar is 10 µm.

**Figure 4:**
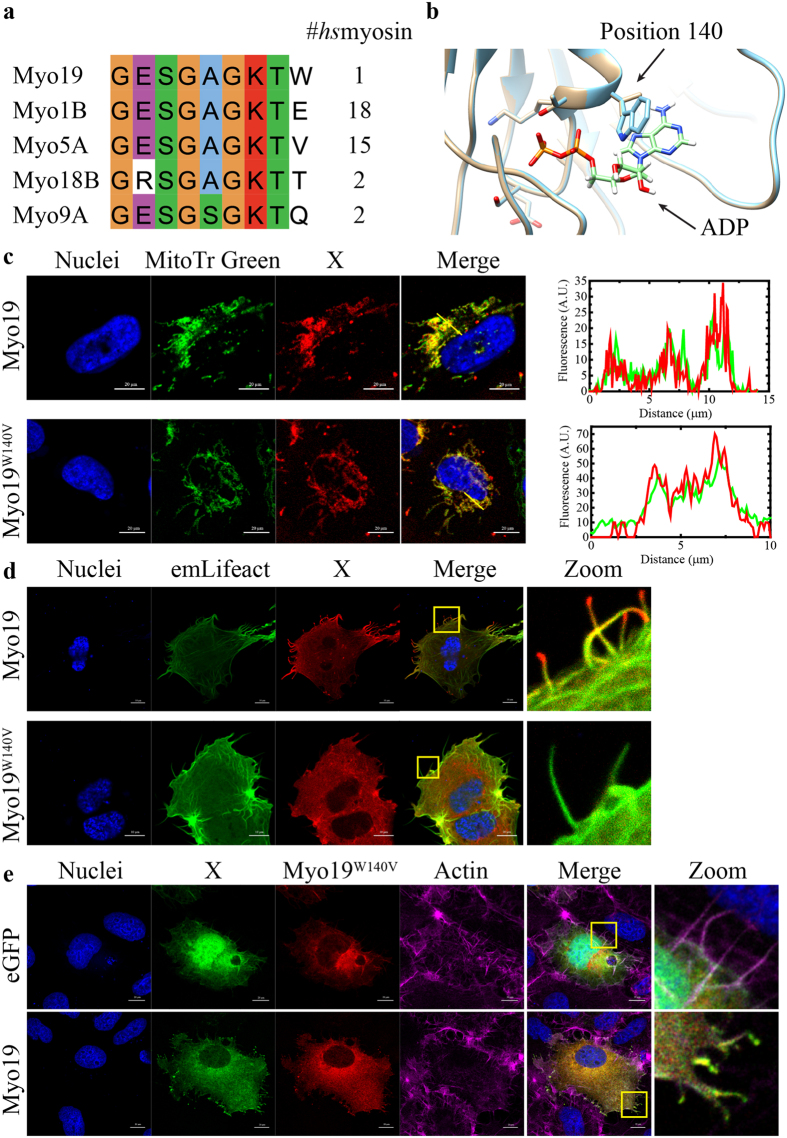
W140 is a conserved residue in Myo19 motor domain which is essential for filopodia tip localization. (**a**) Sequence alignment of human myosin sequences deposited in Swiss-Prot shows the presence of a unique Tryptophan instead of the consensus Valine or Glutamic acid, the unique Tryptophan is preserved in almost all Myo19 homologs (38/39). (**b**) Homology based structure prediction of Myo19 (cyan) and Myo19^W140V^(tan) via the Phyre2 server, aligned with two solved human Myo5 structures (not shown, RCSB: 4ZG4, 1W7J) with bound ADP (shown) in the P-Loop. (**c**) Co-localization of Halo tagged Myo19 or the Myo19^W140V^ mutant with the mitochondrial stain, MitoTracker Green-FM. The intensity plots generated from the yellow line reveal that the mutant localizes to mitochondria similarly to the wild-type. Bar is 20 µm. Blue – nuclei, green – MitoTracker Green-FM stained mitochondria, red – Halo tagged Myo19 or Myo19^W140V^. (**d**) H_2_O_2_ stimulation of U2OS cells over-expressing Halo tagged Myo19 or Myo19^W140V^ results in the localization of Myo19 to filopodia tips, but not of Myo19^W140V^. Bar is 20 µm. Zoom - magnification of the area indicated by the yellow rectangle. The absence of mitochondria is due to them being in a higher focal plane, whereas these filopodia form at the base of the cell. The weak diffuse signal is most likely resulting from Myo19 over-expression or saturation of mitochondria. Blue – nuclei, green – emLifeAct, red – Halo tagged Myo19 or Myo19^W140V^. (**e**) Rescue of Myo19^W140V^ mutant phenotype by WT Myo19. U2OS cells were co-transfected with Halo tagged Myo19^W140V^ and either WT emMyo19 or an eGFP control and induced with H_2_O_2_. As previously shown, Myo19^W140V^ was unable to reach filopodia tips. To our surprise, Myo19 was able to rescue the localization defect and promoted the localization of the mutant to the filopodia tips. Blue – nuclei, green – GFP or emMyo19, red – Halo tagged Myo19^W140V^, Magenta – phalloidin stained actin. Bar is 10 µm.

Next, we considered two competing possibilities: Myo19 localization to the filopodia tip could be coupled to filopodia elongation, or instead occur after the filopodia has formed. To distinguish between these two mechanisms and reveal more about the dynamics of Myo19 localization, we performed time-lapse fluorescence microscopy. Myo19 and actin were visualized by transfecting U2OS cells with full-length Emerald Green–tagged Myo19 and Ruby-tagged LifeAct. We stimulated the cells with 0.2 mM H_2_O_2_ and followed the dynamics of Myo19 and actin over time (Movie S1). Initially, we observed ruffling of the cell membrane, followed by formation of filopodia. The filopodia formation was highly dynamic, with multiple formation and retraction events during the first hour, whereas formation of stable filopodia began after 1 hour. We observed formation of filopodia and subsequent directional movement of Myo19 towards their tips, indicating that Myo19 actively migrates on actin filaments and tracks the tips (Fig. 3a). Several filopodia with Myo19 at their tips were present at the beginning of imaging. Some of the filopodia (2/4) retracted back to the cell, and this was preceded by Myo19 transport to the cell body (Fig. 3b). Overall, these results reveal that Myo19 can move both to filopodia tips and back to the cell body. The forward movement is motor-dependent, as the ATPase-deficient Myo19^G135R^ mutant cannot localize to filopodia tips^20, 28^, and Myo19 is a barbed end–directed myosin^34^. The retrograde motion can be explained by cessation of actin polymerization at the tip and retraction of filopodia back to the cell body with Myo19^39, 40^. Towards the end of the time-lapse movie (~1.5 h), some filopodia seem to be without Myo19 at their tips, but this was most likely due to bleaching, as ~100% of filopodia contained Myo19 at their tips (Fig. 4c). Unfortunately, use of antioxidants to reduce photo-bleaching resulted in inhibition of ROS-induced filopodia formation.

The results of the time-lapse microscopy suggested that the motility of Myo19 towards the barbed end of the actin protrusions is a directional process dependent on the activity of the Myo19 motor. Moreover, if Myo19 only anchored mitochondria to filopodia actin bundles, it would remain in the shaft of filopodia, as the protrusions are elongated by actin polymerization at the tip.

### W140 is a conserved residue in Myo19 motor domain which is essential for filopodia tip localization

The results described above suggested that the enzymatic activity of the motor propels Myo19 and mitochondria towards filopodia tips. Therefore, we searched for amino acids specific to Myo19 that might provide more insights into the enzymology of the motor domain. To identify candidate amino acids, we performed sequence alignment of human Myo19 against other human myosins, as well as against Myo19 homologues from 40 species^41^. We and others^28^ found that W140 is a unique amino acid in Myo19 (Fig. 4a; other myosin classes contain E [18/38] or V [15/38] at this position). This residue is positioned at the edge of the highly conserved P-loop (ATP-binding motif) and at the beginning of the helix preceding Loop 1, a key determinant of several enzymatic properties such as ATPase and motility rate, and nucleotide-binding kinetics^42^. To determine the role of the unique Trp on Myo19, we performed structural homology modeling using the Phyre2 webserver and compared the result with solved myosin structures^43^. The modeling suggested that W140 is in close proximity to the bound nucleotide, and thus might modulate some of the parameters related to nucleotide-binding kinetics (Fig. 4b).

To test the importance of W140, we mutated this residue to a consensus Val and investigated whether Myo19^W140V^ would localize to filopodia tips in response to H_2_O_2_. As with WT Myo19, Myo19^W140V^ localized to mitochondria under normal growth conditions, but failed to localize to H_2_O_2_-induced filopodia tips (Fig. 4c,d). To further assess the importance of W140, we introduced several other point mutations, examining the effect of the residue properties and volume on H_2_O_2_-induced filopodia localization of Myo19. When W140 was mutated to Leu, Glu, Ala, or Gly, the mutant protein failed to show any significant localization to filopodia tips. Myo19^W140F^ was the only mutant indistinguishable from the WT, stressing the critical role of a bulky aromatic residue in position 140 for Myo19 function (Fig. S1). Myo19^W140V^-3IQ, which lacks the C-terminal outer mitochondrial membrane–binding domain, localized to the shaft region of H_2_O_2_-induced filopodia (Fig. S2). This observation strongly indicates that Myo19-3IQ^W140V^ is still capable of actin motility when not bound to the mitochondria, i.e., under no load. To further assess the contribution of motor activity to transport of mitochondria to filopodia tips, we performed rescue experiments by co-overexpressing Myo19^W140V^ with WT Myo19. Expression of the WT protein with the W140V mutant at a 1:1 ratio resulted in localization of both myosins to filopodia tips, indicating that the WT protein rescued the mutant phenotype, and strongly supporting that Myo19 functions as an ensemble of motors (Fig. 4e).

This result also indicated that the Myo19^W140V^ mutant does not act in a dominant-negative manner when in stoichiometric proportions with the WT. The underlying mechanism for the inability of Myo19^W140V^ to transport the mitochondria as a cargo could be understood by studying its ATPase cycle to reveal key enzymatic properties that were lost or modified. To this end, we co-expressed and purified the motor domain with the lever arm consisting of three IQ calmodulin-binding motifs, as performed previously for the WT (Myo19-3IQ and Myo19^W140V^-3IQ, Fig. S3).

### Steady-state ATPase activity reveals alterations in Myo19^W140V^ enzymology

**Figure 5:**
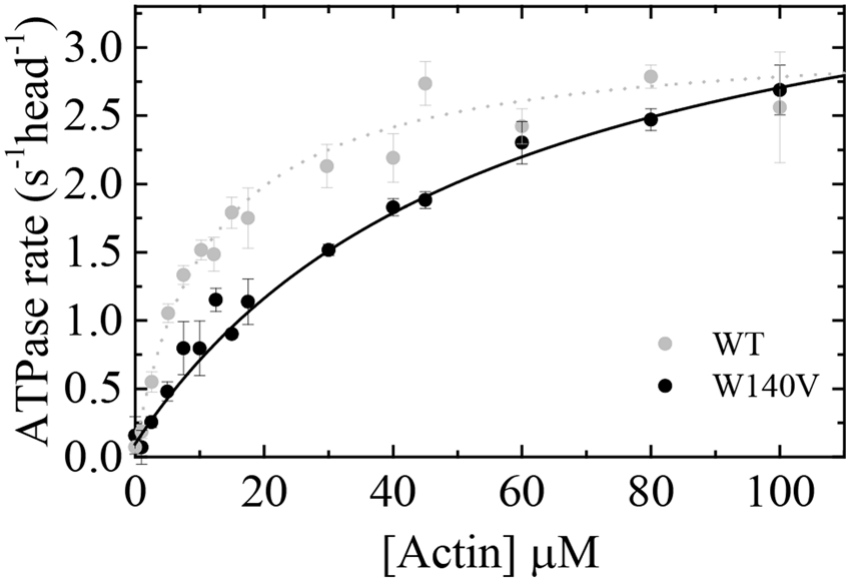
Actin activated steady state ATPase activity reveals alterations in Myo19-3IQ^W140V^ enzymology. The actin filament concentration dependence of the Myo19-3IQ (WT) and Myo19-3IQ ^W140V^ (W140V) steady-state ATPase activity. The solid line through the data points is the best fit to a rectangular hyperbola ($v={v}_{0+}({k}_{cat}[actin])/({K}_{ATPase}+[actin])$). Error bars represent standard deviation from at least three independent experiments from different protein purifications. Data for Myo19-3IQ (WT) is reproduced from (Usaj & Henn, parallel submission).

To determine whether the W140V point mutation had any effect on Myo19^W140V^ enzymology, we measured the actin-dependent ATPase steady state activity of Myo19^W140V^-3IQ (Fig. 5). The steady-state parameters of Myo19^W140V^-3IQ revealed that, although the mutant’s *k*_cat_ (ATP turnover per myosin head) was slightly faster than the WT’s, its *K*_ATPase_, (the actin concentration at the half maximum the ATPase activity), was significantly (5-fold) weaker (Table 1). This suggests that the intrinsic rate constants of Myo19^W140V^-3IQ ATPase cycle were significantly affected by the point mutation.

**Table 1:** Kinetic parameters comparison between Myo19-3IQ and Myo^W140V^-3IQ.

Parameter	Value	
	Myo19-3IQ^5^	Myo19-3IQ^W140V^
**Steady-state ATPase parameters**		
*k*_cat_ (s^−1^ head^−1^)	3.1 ± 0.1	4.1 ± 0.4
*K*_ATPase_ (µM)	11.6 ± 1.2	56.7 ± 10.9
*v*_o_ (s^−1^)	0.06 ± 0.02	0.10 ± 0.05
**ATP binding**		
*1*/*K*′_1T_ (µM)	476 ± 116	758 ± 217
*K*′_+2T_ (s^−1^)	1003 ± 26	381 ± 72
*K*′_1T_ *K*′_+2T_ (μM^−1^s^−1^)	2.17 ± 0.8	0.5 ± 0.2
*K*′_−2T_ (s^−1^)	≈0	1.4 ± 0.3
*k*_+α_ (s^−1^)	17.5 ± 0.3	29.3 ± 4.3
*k*-_α_ (s^−1^)	2.6 ± 1.1	10.1 ± 6.0
*K*_α_^3^	6.8 ± 1.6	2.9 ± 1.3
**ADP binding**		
*K*′-_1D_ (s^−1^)	9.3 ± 0.3	12.7 ± 1.5
*1*/K_D,overall_ (µM)	0.13 ± 0.04	1.6 ± 0.3

Conditions: 20 mM MOPS, pH 7.3, 50 mM KCl, 2 mM MgCl_2_, 0.2 mM EGTA, 1 mM DTT, 25 °C. ^1^Calculated parameter from rates or/and equilibrium constants. ^2^Calculated parameters from y-intercept. ^3^The equilibrium constant for isomerization, *K*_*α*_, is define as *K*_*α*_ = *k*_+__*α/*_ *k*_−*α*_. ^4^Calculated parameters from *k-*_*α*_ = *k*_+__*α/*_ *K*_*α*_. ^5^Reported in (Usaj & Henn, parallel submission).

**Figure 6:**
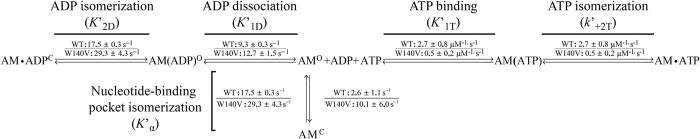
Kinetic reaction scheme comparing the key biochemical transitions between Myo19-3IQ (WT) and Myo19-3IQ^W140V^ (W140V) mutant. In this scheme, four nucleotides related kinetic transitions are shown: ADP isomerization step, ADP release step, ATP binding and nucleotide binding pocket isomerization. The rate constants are also listed in Table 1. WT and W140V are abbreviation for the Myo19-3IQ and Myo19-3IQ^W140V^ mutant, respectively.

In this study, we present the kinetic analysis of Myo19^W140V^-3IQ within the framework of the WT Myo19-3IQ ATPase cycle that is fully characterized in the companion paper (Ušaj & Henn, parallel submission). The key rate and equilibrium constants that are compared between the WT and mutant protein are presented in Table 1. In the following sections, we present our analysis of ATP and ADP binding according to the nomenclature shown in the kinetic scheme, Fig. 6. The kinetic scheme in Fig. 6 shows the determined rate and equilibrium constants of both protein constructs. Myo19^W140V^-3IQ construct did not exhibit any FRET signal from the tryptophan(s) (λ_ex, 280_ nm) to the fluorescent mant moiety (λ_em,>400_ nm) upon binding of mant nucleotides, whereas the WT exhibits such signal (Fig. S4). The loss of signal upon mant nucleotide binding limited the measurements of the mutant to direct measurements of nucleotide binding. However, this is quite interesting since it clearly shows that position 140 is very sensitive to nucleotide binding. Thus, it may have an effect on the interaction of Myo19 with nucleotide binding despite being situated between, and not within, the two-well-known nucleotide interacting motifs, P-loop and Loop I.

### ATP binding to Acto·Myo19^W140V^-3IQ occurs via the same mechanism as the WT, but with modulated rates

**Figure 7:**
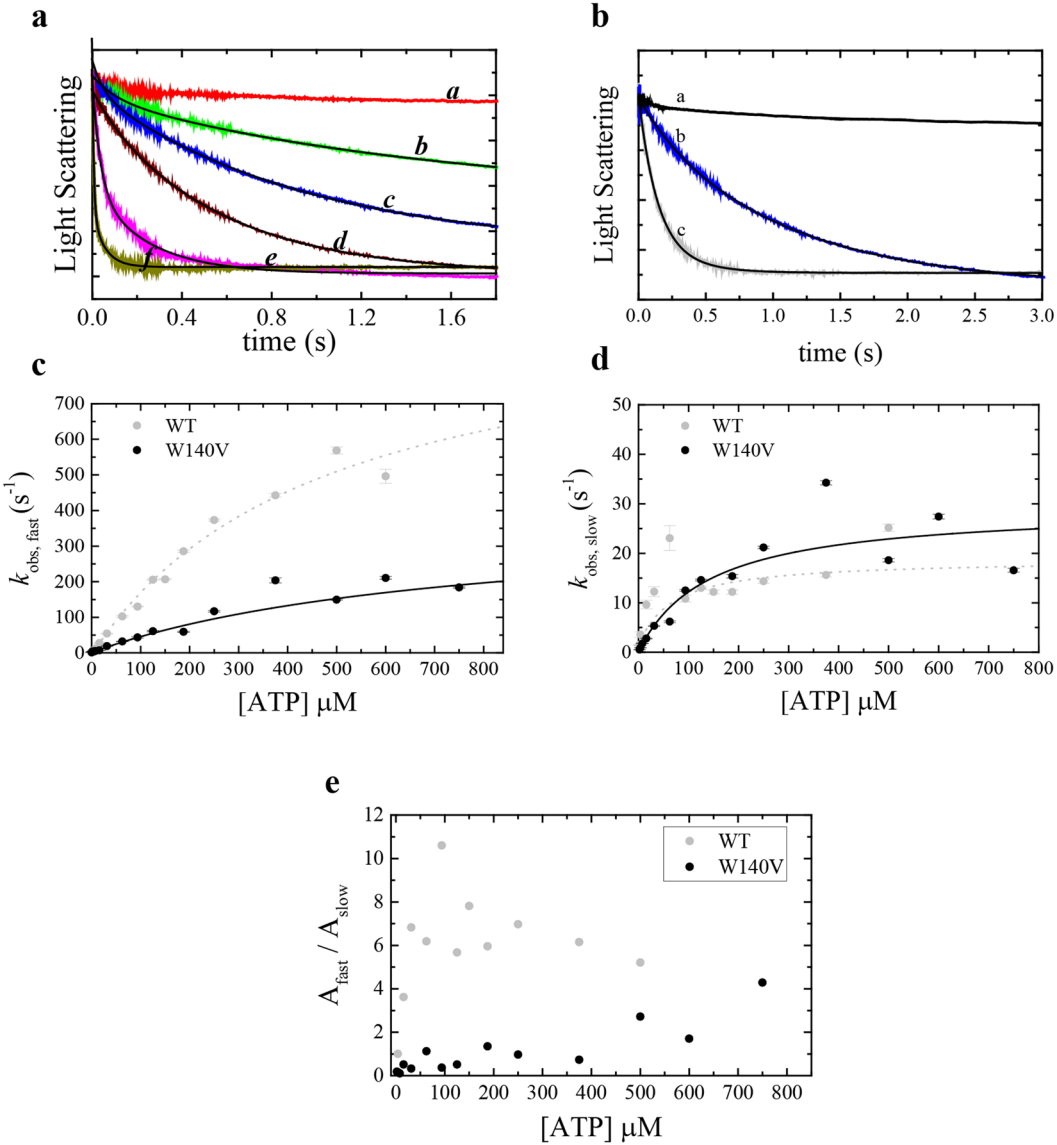
ATP binding to Acto·Myo19^W140V^-3IQ (W140V) shows different kinetic behavior than the Myo19-3IQ (WT) (**a**). Time courses of light scattering decrease after mixing 0.25 µM actomyosin with 0 µM (**a**), 1.95 (**b**), 3.9 (**c**), 7.8 (**d**), 62.5 (**e**), 125(f), µM ATP for Acto·Myo19^W140V^-3IQ. Data are averaged transients (n = 3–5). The black lines are the best fits to double exponential function (see SI for analysis of the residuals of the fits). (**b**) Time course of light scattering decrease after mixing 0.25 µM actomyosin with 0 µM (**a**) or 7.8 µM ATP for Acto·Myo19-3IQ^W140V^ (**b**) or Acto·Myo19-3IQ (**c**). Data are averaged transients (n = 3–5). The smooth lines through the data represent best fit to double exponential function (**c**). [ATP]-dependence of the fast-observed rate constant of ATP binding to Acto·Myo19-3IQ^W140V^ in comparison to Acto·Myo19-3IQ as measured by light scattering. (**d**) [ATP]-dependence of the slow observed rate constant of ATP binding to Acto·Myo19-3IQ^W140V^ in comparison to Acto·Myo19-3IQ as measured by light scattering. The lines through the data points in **c** and **d** are the best fit to rectangular hyperbola. Error bars of the fitting are within data points. (**e**) The ratio of the fast (A_fast_) to slow (A_slow_) amplitudes of the observed fast and slow rate constants as a function of [ATP] for Acto·Myo19-3IQ^W140V^ in comparison to Acto·Myo19-3IQ. Data for Myo19-3IQ (WT) is reproduced from (Usaj & Henn, parallel submission).

We measured ATP binding to Acto·Myo19^W140V^-3IQ by monitoring actin dissociation upon ATP binding. Similarly to WT Myo19-3IQ, but in contrast to many other studied myosins, Myo19^W140V^-3IQ binding to F-actin labeled with common actin-fluorescent probes (i.e., pyrene, coumarin, or IAEDANS) did not yield any signal. Therefore, we monitored ATP binding to Acto·Myo19^W140V^-3IQ by measuring changes in light scattering. ATP binding to Acto·Myo19^W140V^-3IQ induced dissociation from actin (Fig. 7a), exhibiting double-exponential kinetics similar to those observed for the WT (Figs 7b, S5). The fast phase is due to rapid ATP binding to readily accessible actomyosin conformation complex, while the slow phase results from the isomerization of the nucleotides binding pocket from the ‘close’ (AM^C^) state to the ‘open’ (AM°) state preceding ATP binding. *K*′_α_ is the isomerization equilibrium constant described by the slow phase. The fast and slow observed rate constants (*k*_obs,fast_, *k*_obs,slow_) of Acto·Myo19^W140V^-3IQ exhibited hyperbolic dependence on [ATP] (Fig. 7c,d). Thus, the mechanism of ATP binding to Acto·Myo19^W140V^-3IQ is the same for both the mutant and the WT, with isomerization of Acto·Myo19^W140V^-3IQ preceding ATP. The ATP-binding mechanism is presented in more details in our co-submitted paper (Ušaj & Henn, parallel submission). Despite having a similar binding scheme, the rate and equilibrium constants for ATP binding to Acto·Myo19^W140V^-3IQ were strongly altered in comparison with those of WT-Myo19-3IQ (Table 1). Fitting the data of the mutant’s *K*′_obs,fast_ yielded an equilibrium constant (^1^/*K*′_1T_) for ATP binding of 758 ± 217 µM, with a *K*′_+2T_ (maximum rate of isomerization) (*K*′_+2T_ ≈ *K*′_diss_) to the dissociated state of 381 ± 72 s^−1^. The fit of the curve for the mutant Myo19 is distinct from the origin (*K*′_−2T_ = 1.4 ± 0.3), suggesting that ATP binding is slightly reversible compared with that observed for the WT (*K*′_+2T_» *K*′_−2T_ ~0) (Fig. 7c). The *k*_obs,fast_ hyperbolic dependence on [ATP] yields a second-order association rate constant for ATP binding of Acto·Myo19^W140V^-3IQ (*K*′_1T_ *K*′_+2T_ = 0.5 ± 0.2 µM^−1^·s^−1^) that was approximately 4-fold smaller than that of the WT (Table 1). Overall, the rate constants describing ATP binding are significantly different between the WT and the mutant Myo19-3IQ constructs (Table 1), indicating that the W140V mutation strongly affects ATP-binding kinetics.

The slow phase observed upon ATP binding exhibited hyperbolic dependence on [ATP] (Fig. 7d). As with the WT, the slow phase arises from an isomerization between the “open” (AM°) and “closed” (AM^C^) nucleotide-binding states. Notably, the hyperbolic dependence of the slow phase was more pronounced for the mutant than for the WT. In this regard, the mutant resembles Myo1c, in which the slow phase has larger amplitudes and exhibits clear hyperbolic dependence on [ATP]. Overall, the maximal *k*_obs, slow_ = *K*′_+α_ for Myo19^W140V^-3IQ (29.3 ± 4.3 s^−1^) was 2-fold faster than that for the WT (Fig. 7d). The ratio of the amplitudes of the fast and slow phases (A_fast_/A_slow_) defines the equilibrium constant *K*_α_ for the AM° to AM^C^ states^42^. To estimate the mutant’s *K*_α_, we averaged the A_fast_/A_slow_ ratios at the three highest [ATP] values tested, based on a previously reported approach^44^. The *K*_α_ of the WT was 2-fold larger than that of the mutant (6.8 ± 1.6 versus 2.9 ± 1.3, respectively); hence the AM^C^ state was more populated in the mutant than in the WT. As [ATP] increased, the observed transients of ATP binding to Acto·Myo19^W140V^-3IQ were dominated by the fast phase (Fig. 7e). This is because the product of *k*_+1T_ × [AM°] × [T] is much greater than *K*′_+α_, resulting in the faster depletion of the AM° state at high [ATP] than its replenishment by isomerization of the AM^C^ to AM° state, with a maximal rate of *K*′_+α = _29.3 ± 4.3 s^−1^ (Table 1). Finally, knowledge of *K*′_α_ and *K*′_+α_ permitted us to calculate *K*′_−α_, which was ~4-fold faster for the mutant (10.1 ± 6.0 s^−1^) than for the WT (Table 1).

### W140V substitution enhances ADP-binding transitions in comparison with the WT

**Figure 8:**
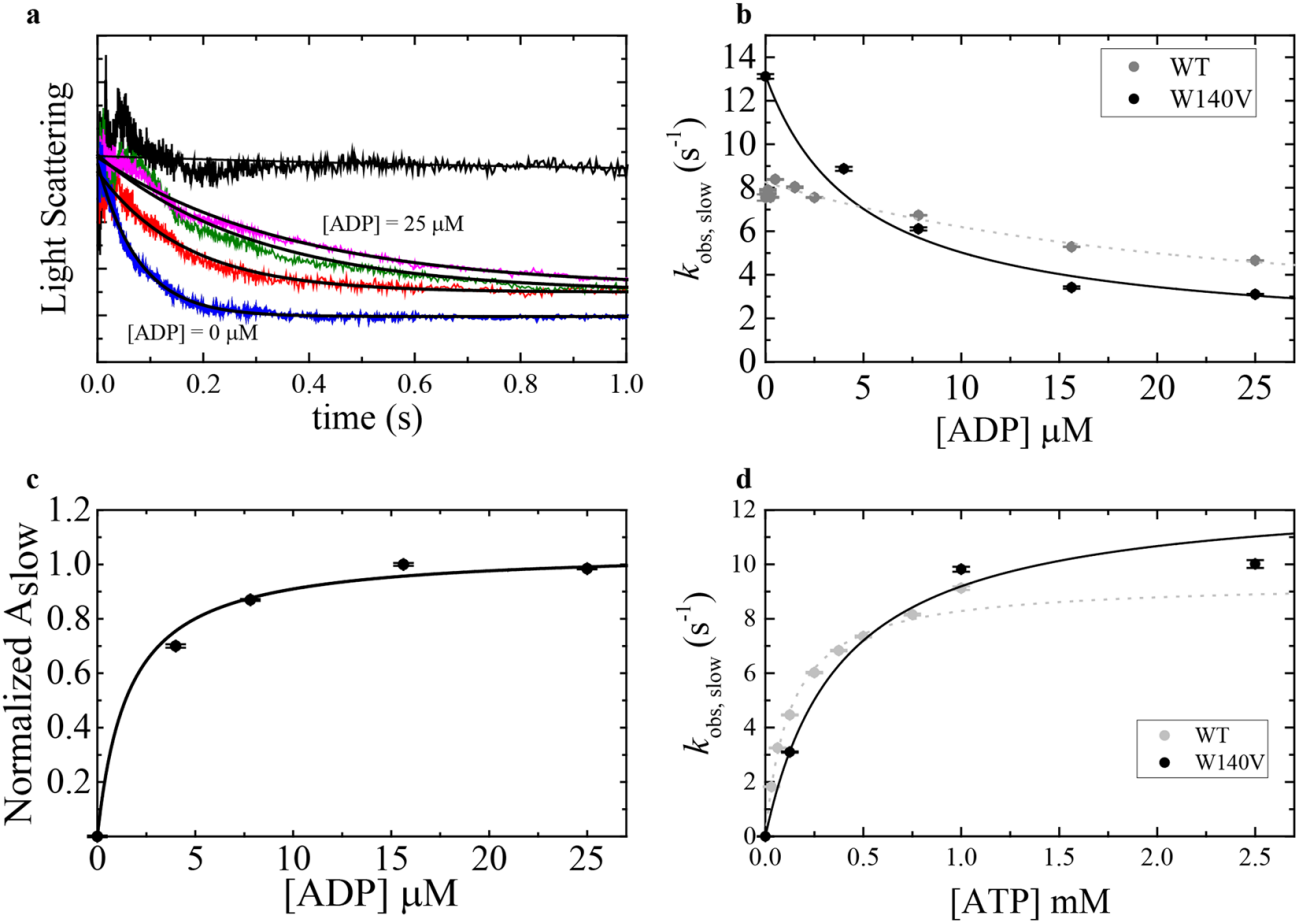
ADP dissociation from *Acto·Myo19-3IQ* ^*W140V*^ (W140V) measured by kinetic competition with ATP in comparison to *Acto·Myo19-3IQ* (WT). (**a**) Normalized time courses of light scattering decrease after mixing 0.2 µM Acto·Myo19-3IQ^W140V^ with 0 µM ATP (upper trace) or 125 µM ATP in presence of different [ADP] ranging from 0 to 25 µM. Data are averaged transients (n = 3–5). Smooth lines (black) through the data represent best fits to double or single exponential functions. (**b**) [ADP]-dependence of the observed slow rate constant for Acto·Myo19-3IQ and single observed rate constant for Acto·Myo19-3IQ^W140V^ as measured by kinetic competition with 125 µM ATP. (**c**) [ADP]-dependence of normalized amplitudes of the slow phase obtained by fitting transients described in (**a**). (**d**) [ATP]-dependence of the observed rate constant for Acto·Myo19-3IQ and Acto·Myo19-3IQ^W140V^ dissociation as measured by light scattering at saturated [ADP] of 25 µM. The lines through the data points in (**b**,**c** and **d**) are the best fits to rectangular hyperbola. Error bars of the fittings are within data points. Data for Myo19-3IQ (WT) is reproduced from (Usaj & Henn, parallel submission).

The rate of ADP release and ADP’s affinity towards actomyosin can be determined by measuring the rate of ATP-induced dissociation of myosin from actin as a function of increasing prebound ADP. Such transients exhibit double exponentials, with the fast phase reporting ATP binding to the fraction of ADP-free actomyosin and the slow phase reporting the release of ADP. The amplitudes of the fast phase decrease as [ADP] increases until the fast phase is no longer observed, and actomyosin dissociation exhibits only a single (slow) phase. The observed rate constants for Acto·Myo19-3IQ^-W140V^ ADP release were similar to those for WT Acto·Myo19-3IQ·ADP (Usaj&Henn), despite having a much lower signal. Figure 8a shows time courses of Acto·Myo19-3IQ^-W140V^·ADP upon mixing with excess ATP (125 µM). The collection of data for the mutant was very challenging due to the weak signal; consequently, Fig. 8a shows only limited time courses. Nevertheless, we can extrapolate from the observed rate constants to obtain the slow-phase rate of *k*_obs,slow_ ≈ 2.9 s^−1^, which differs only slightly from that of the WT (Fig. 8b). The affinity of ADP for Acto·Myo19-3IQ^-W140V^ is determined from the hyperbolic dependence of the amplitudes of the slow phase (A_slow_) versus [ADP]^45^, and can be fitted according to Equation 1:

1 $${{\rm{A}}}_{{\rm{slow}}}=\frac{[{\rm{ADP}}]}{\frac{1}{K{^{\prime} }_{D}}+[{\rm{ADP}}]}$$

The W140V mutation had a 10-fold weaker ADP affinity than the WT (1/*K*′_D_ = 1.6 ± 0.3 µM; Fig. 8c, Table 1), but nonetheless maintained a relatively strong affinity towards ADP. Myosin classes with high affinities towards ADP require much higher [ATP] for examination of the true ADP dissociation rate constant^46^. Therefore, we performed ATP-induced dissociation of Acto·Myo19^W140V^-3IQ in the presence of saturating [ADP] = 25 µM with increasing [ATP]. The *k*_obs_ of the single exponential phase exhibited hyperbolic dependence on [ATP], saturating at *K*′_−1D_ = 12.7 ± 1.6 s^−1^, which is a bit faster than that of the WT (Fig. 8d, Table 1), but in accordance with a faster *k*_cat_ observed for the mutant.

WT Acto·Myo19-3IQ·ADP dissociation at saturated [ADP] exhibited a well-resolved lag upon mixing with ATP (Ušaj & Henn, parallel submission). By contrast, Acto·Myo19^W140V^-3IQ·ADP exhibited a much shorter lag phase, even at lower temperatures. This suggests that the mutation has a mild effect on ADP binding and dissociation kinetics (*k*_+1D_, *k*_*−*1D_, Fig. 6), but a more pronounced effect on isomerization kinetics (*k*_+2D_, *k*_*−*2D_, Fig. 6).

### Conserved mechanism for nucleotide binding for both the mutant and the WT

Similarly to WT Myo19, the mutant’s ADP release was faster than the observed *k*_cat_ but in the range to significantly contribute to the catalytic turnover of the ATPase cycle of Myo19^W140V^; hence the overall mechanism is conserved (Ušaj & Henn, parallel submission). Comparison of the WT and mutant nucleotide binding and isomerization steps revealed that at least two transitions limit the ATPase cycle under no external load (Fig. 6). Using the rates for ADP release (Fig. 6), the steady-state ATPase rate (*k*_cat_) for the mutant would be ~8.6 s^−1^ head^−1^, twice as fast as our measured *k*_cat_ (4.1 s^−1^ head^−1^). Because a similar ratio was observed for WT (Ušaj & Henn, parallel submission), we argue in favor of the existence of an additional isomerization step (AMD^C^ to AMD°) before ADP is completely released. Thus, the mutation inhibited Myo19 biological function by altering the intrinsic kinetic rate constants. The faster observed ADP transitions for Myo19^W140V^ suggest lower efficiency of cargo translocation. This may be a consequence of the shorter dwelling time in the load-bearing ADP states; however, this proposal needs to be tested by force measurements and/or single-molecule experiments in the future.

### The altered kinetics of Myo19^W140V^-3IQ impact its duty ratio

**Figure 9:**
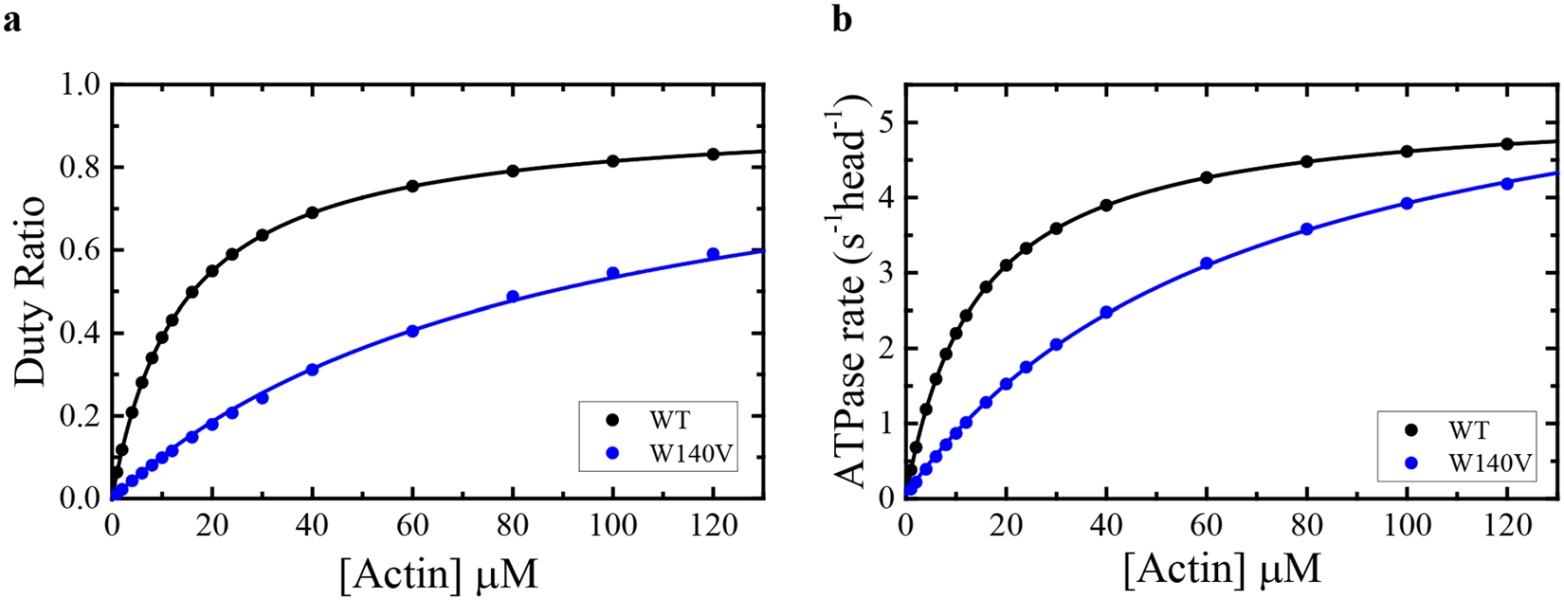
Simulation of the ATPase activity and duty ratio of Myo19-3IQ (WT) and Myo19^W140V^-3IQ (W140V). (**a**) Simulated steady state [actin]-dependence of the ATPase activity of the Myo19-3IQ and the mutant Myo19^W140V^-3IQ. The solid lines through the data points are the best fit to a rectangular hyperbola ($v={v}_{0+}({k}_{cat}[actin])/({K}_{ATPase}+[actin])$). (**b**) Duty ratio as a function of [actin]. The duty ratio was calculated from the summation of all the biochemical intermediates distribution at each [actin] at the steady-state time regime according to equation: duty ratio = (strongly bound states)/(strongly bound states + weakly bound states). The solid lines through the data points are the best fit to a rectangular hyperbola ($duty\,ratio=1\ast [actin])/({K}_{DR}+[actin])$). Data for Myo19-3IQ (WT) is reproduced from (Usaj & Henn, parallel submission).

The simulated ATPase cycle for WT permitted us to integrate the modified equilibrium and rate constants that we measured for Myo19^W140V^-3IQ, and to test whether they are sufficient to account for the observed weaker *K*_ATPase_ and a slightly faster *k*_cat_ for the mutant. We found that weakening of *k*_+AP*i*_ (defined as the second order rate constant for MADP·P_*i*_ binding to actin) by 6-fold compared with the WT resulted in *K*_ATPase_ and *k*_cat_ of 65.0 ± 0.7 µM (measured 56.7 ± 10.9 µM) and 6.5 ± 0.02 s^−1^ head^−1^ (measured 4.1 ± 0.4 s^−1^ head^−1^; calculated from the two slowest phases of ADP isomerization and its release ≈8.6 s^−1^ head^−1^), respectively. The *K*_DR_ (the [actin] at 0.5 duty ratio) for Myo19^W140V^-3IQ was 92.7 ± 0.6 µM, suggesting that the changes in the measured and estimated rates could account for the mutant motor’s ~7-fold weaker *K*_DR_ (Fig. 9). Similarly, the duty ratio can be calculated according to De La Cruz *et al*.^47^ by the following parameters: *k*_+APi_, *K*_H_, and *K*_−D_ (~0.15 µM^−1^ s^−1^, 1.07 and 8.6 s^−1^, respectively), the *K*_DR_ is 111 µM. Myo19^W140V^-3IQ will require an actin concentration of ~400 µM to reach a duty ratio of ~0.8 under saturating [ATP], concluding that Myo19^W140V^-3IQ is a very low duty ratio motor.

## Discussion

Our results show that the starvation-induced localization of Myo19 to filopodia is mediated by ROS, and that Myo19 can be recruited to filopodia tips by direct addition of H_2_O_2_ to the media. We also showed that mDia2ΔDAD, a factor that induces filopodia growth, could promote a similar localization of Myo19 to filopodia tips. By contrast, Myo10, which also promotes filopodia formation, failed to trigger Myo19 localization to filopodia. These observations suggest that Myo19 activation can be mediated by both intracellular and extracellular signals. Time-lapse microscopy revealed that Myo19 pauses during the formation and growth of filopodia prior to tracking their tips, and can also undergo retrograde movement coupled to filopodia retraction. Finally, we connected the enzymology of Myo19 to its filopodial activity by studying in detail the enzymology of a class-specific mutation in a highly-conserved residue, W140. We propose that modulation of the duty ratio of the mutant is partially responsible for its inability to perform the filopodia transport activity.

Glucose starvation increases cellular ROS levels and promotes a specific phosphorylation pattern involving focal adhesion proteins^29^. We hypothesized that the filopodial localization of Myo19 is mediated in a similar way. The inhibition of glucose starvation–induced filopodia localization by extracellular catalase and the synergistic effect of PG indicated that cells excrete ROS into the environment, which then enter the cell and exert their effects^29^. The mimicking of this phenotype by direct addition of H_2_O_2_ supports this mechanism. ROS participate in and affect signaling pathways by altering the activity of kinases or inhibiting phosphatases, and may directly modify Myo19 or alter the activity of an interacting protein^48, 49^. For example, EGF signaling involves cascades of kinase phosphorylation, and it is well established that EGF stimulation leads to filopodia formation^50, 51^. Thus, such signaling pathways may be also linked to Myo19 filopodial activity.

The majority of the motility we observed is directional and persistent towards the filopodia tips. This suggests a possible regulatory mechanism mediated either by affecting the motor’s ability to propel mitochondrial movement or by additional motility licensing factors that are regulated during filopodia elongation. The movement of Myo19 along the filament tracks appears to be deterministic, and is most likely carried out by an ensemble of motors. This is supported by the fact that we can observe motility of foci that must consist of more than a single fluorophore arising from tagged Myo19, which can be easily observed when they wrap around an organelle such as the mitochondria. Additional evidence supporting the idea that Myo19 acts through an ensemble of motors is provided by the observation that co-expression of WT Myo19 and the mutant Myo19^W140V^ rescued the mutant phenotype, resulting in the localization of both WT and mutant motors at filopodia tips. This may arise from the presence of both WT and mutant motors on the same membrane, or from some as-yet-unknown interaction between several Myo19 motors.

Several other myosins localize to filopodia and affect their formation, likely by supplying the necessary components for filopodia formation. These myosins bring various cargos to the filopodia, such as actin bundling proteins, and proteins involved in cell adhesion such as β-integrins and VE-Cadherin^52–56^. Myo10 possesses unique forward and rearward movements within the filopodia of HeLa cells and promotes filopodia formation when ectopically expressed^35, 57–59^. Dictyostelium Myo7 fulfills a function similar to that of Myo10 within the filopodia^56, 59, 60^. Myo3 brings Espin1, an actin bundler, to filopodia and promotes their elongation^54, 61^. A barbed end–directed mutant of Myo6 can also promote filopodia formation, likely via the bundling activity of the dimer^62^. Myo15 localizes to filopodia tips, and Myo15-null mice have short stereocilia in comparison with WT animals^63^.

Myo19 and Myo19^W140V^ pose a great challenge for *in vitro* characterization, mainly because of their lack of quenching of fluorescent actin, as well as their silent fluorescence upon ATP and ADP binding. However, the clear cellular phenotype allows us to test how the motor enzymology supports the protein’s activity. Most importantly, we were able to identify key parameters in the ATPase cycle of Myo19^W140V^ that may account for the observed phenotype. First, the W140V mutant had a slightly higher *k*_cat_ but a ~5-fold weaker *K*_ATPase_ in comparison with the WT. Second, both the *k*_α_ transition (AM^C^ ↔ AM°), and the *k*_αD_ transition (AMD^C^ ↔ AMD°) were faster in the mutant. Third, the lag preceding ADP release was much more prominent in the WT than in the mutant. Overall, these changes indicate that, although the mechanism remained similar, the rate and hence the equilibrium constants of the biochemical transitions became faster in the mutant. Because we established a strong link between the *K*_ATPase_ and the *k*_+AP*i*_ of Myo19, we can model the entire ATPase cycle of Myo19^W140V^-3IQ. Despite not having all the cycle parameters at hand, we predict that the *K*_DR_ of the mutant was reduced by ~7-fold compared with the WT. It is reasonable to speculate that a multi-motor ensemble of Myo19 on the mitochondria would compensate for the reduction in the duty ratio of the motors, especially in a dense actin mesh. However, it is also reasonable to assume that unique load-dependence transitions are also important for this motor function; hence the weakened ADP affinity may also affect the force-sustained states. Previous authors have proposed that myosins that undergo the *K*_α_ transition are highly force-sensitive^64^. Studies performed on Myo1c on the effect of R156W mutation which has been shown to increases *K*_α_^65^ may suggest some parallel link to explain how W140V is hindering Myo19 transport of mitochondria. The frictional loading assays indicated that load-induced inhibition of actin gliding is less pronounced in the R156W Myo1c mutant^65^. It has been proposed that an increase in *K*_α_ (i.e., a stabilization of the nucleotide-accessible AM° state) might be an indication of reduced tension sensitivity^65^. If this hypothesis is true, then in our case the W140V mutation (decreases *K*_α_) may produce a Myo19 motor with higher sensitivity to load. Such motors would translocate cargo less efficiently. Future work should seek to test this hypothesis.

The physiological role of mitochondria in filopodia formation and elongation remains unknown. Recent studies show that mitochondria migrate into damaged axons, and that a high density of mitochondria in a damaged axon is correlated with improved regeneration. When an axon regenerates, it forms a growth cone, which is an actin-based structure whose size is correlated with the density of mitochondria within it. Collectively, these studies reveal that the major role of the mitochondria in the damaged axon is to produce ATP that is essential for repair, possibly by replenishing the local pool of G-Actin–ATP, which is essential for filopodia elongation by formins^66–68^. In addition, axon branching occurs preferentially near stalled mitochondria, and the latter act as hot spots of protein synthesis^69^. These remarkable findings suggest that mitochondrial motility and localization is strongly coupled to ATP production and actin dynamics. We propose that Myo19 plays a similar role, increasing mitochondrial density to supply ATP at filopodia tips.

## Materials and Methods

### Reagents

All chemicals and reagents were of the highest purity commercially available. ATP was purchased from Roche Applied Science (Penzberg, Germany) and ADP was purchased from Bio Basic (Markham, ON, Canada). Nucleotide concentrations were determined by absorbance at 259 nm using ε_259_ of 15,400 M^−1^ cm^−1^. In all experiments one molar equivalent of MgCl_2_ was added to nucleotide solutions immediately before use.

3-(N-Morpholino)propanesulfonic acid (MOPS), Ethylene glycol-bis(2-aminoethylether)-N,N,N’,N’-tetraacetic acid (EGTA), Apyrase (potato grade VII), phalloidin were purchased from Sigma-Aldrich (St. Louis, Missouri, USA). Alternatively, the same form of phalloidin was purchased also from Setareh Biotech (Eugene, OR, USA). MgCl_2_∙6H_2_O came from Bio Basic (Markham, ON, Canada) and KCl from Merk (Darmstadt, Germany).

### Cloning of Myo19 constructs

Emerald tagged Myo19 (emMyo19) and Ruby tagged Myo19 (Myo19-Ruby) were generated by swapping the eGFP from Myo19-eGFP^20^ with Emerald or Ruby from mEmerald-Lifeact-7 or mRuby-LifeAct, respectively, using the restriction enzymes AgeI and NotI. emMyo19, full length Myo19-Halo, or Myo19-3IQ-Halo^20^ (Usaj and Henn, parallel submission) were used as templets for generation of Myo19 mutants and Myo19-3IQ mutant constructs (Table S1). For expression and purification, Myo19^W140V^-3IQ was cloned into the pF14A Flexi Vector using the restriction enzymes NheI and PspXI (Promega). Human calmodulin (Myo19 light chain according to^18^) was cloned to pF4A CMV Flexi Vector (Promega) according to the manufacturer’s protocol. mEmerald-Lifeact-7 was a gift from Michael Davidson (Addgene, Cambridge, MA, USA, plasmid # 54148). Plasmid expressing Myo10 was a kind gift of Prof. Alexander Bershadsky (MBI, NUS). eGFP-tagged full-length mDia2/Drf3 was a kind gift from Drs. Klemens Rottner and Jan Faix (TU Braunschweig and HMS, Germany, respectively). mRuby-LifeAct was a kind gift from Prof. Arie Admon (Technion - IIT, IL). Primers pairs harboring a mutation (Table S1) were phosphorylated using T4 PNK (NEB, Ipswich, MA, USA) according to the manufacturer’s instructions. The primers were used to amplify the template using Phusion High fidelity DNA polymerase (Thermo Fisher Scientific, Waltham, MA, USA). Ligations using T4 ligase (Thermo Fisher Scientific, Waltham, MA, USA) were carried out according to the manufacturer’s instructions.

### Cell culture reagents

All materials were purchased from Sigma unless otherwise indicated. Fetal calf serum, L-Glutamine, HEPES-KOH pH 7.4, penicillin, streptomycin, amphotericin B were purchased from Biological Industries, Beit Haemek, Israel. Paraformaldehyde (PFA) was purchased from EMS (Hatfield, PA, USA).

### Cell culture, cell lines, starvation conditions and antioxidants

U2OS cells were grown at 37 **°**C and 5% CO_2_ in DMEM supplemented with 10% Fetal calf serum (FCS, Biological industries, Beit Haemk, Israel), 4.5 g/L glucose, 2 mM L-Glutamine, 20 mM HEPES-KOH pH 7.4, 100 U/ml penicillin, 100 µg/ml streptomycin and 0.25 µg/ml amphotericin B. Starvation conditions: Cells were rinsed twice with PBS and incubated in starvation medium (Glucose free DMEM supplemented with 20 mM HEPES-KOH pH 7.4, 5 mg/ml BSA, 100 U/ml penicillin, 100 µg/ml streptomycin and 0.25 µg/ml amphotericin B) for two hours. Antioxidants were prepared according to the manufacturer’s directions and diluted in media prior to being added to the cells.

### Immunofluorescence

Cells grown on coverslips were fixed with 4% PFA in CBS (10 mM MES pH 6.1, 138 mM KCl, 3 mM MgCl_2_, 2 mM EGTA, 7.8% sucrose) for 10 min. The PFA was removed by washing with 0.3% TBST (0.3% Tween-20 in Tris buffered saline), and the cells were blocked for one hr with 10% BSA in TBST. Primary antibody was added at a dilution of 1:250 in TBST for one hr (Myo19 -Abcam ab174286, ATP5a – Abcam ab14748). The coverslips were washed three times for five mins in TBST and a secondary antibody was added at a dilution of 1:250 in TBST for one hour (Alexa-fluor 488 conjugated anti rabbit (Life Technologies) and Alexa fluor 633 conjugated anti mouse (Life technologies). The coverslips were washed three times for five mins in TBST (once in the presence of Hoechst) and mounted on slides using Fluoromount-G. Actin in fixed samples was visualized by incubating the cells with either Phalloidin 633 or Phalloiding 546 (Life technologies).

### Transfections and microscopy

Transfections were performed using Polyethylenimine (PEI) purchased from Weis Scientific – PolySciences (Warrington, PA, USA). Adherent U2OS cells were plated a day before transfection on glass bottom dishes purchased from *In-vitro* Scientific (Mountain View, CA, USA) and allowed to adhere overnight. Plasmid DNA and PEI were diluted separately in 150 mM NaCl, combined and complex formation was allowed for 25 min at RT before addition to the cells and incubation overnight. Hoechst 33342 (0.75 µg/ml) was added 15 min prior to imaging. To visualize Halo tagged Myo19, HaloTag TMR ligand (Promega) was added to cells transfected with Halo tagged Myo19 at a final concentration of 50 nM and incubated overnight. Images were taken using LSM710 at ×40 magnification or INCELL2000 at ×60 for live cells in an environmental chamber or using LSM710 at ×63 for fixed samples.

### Microscopy settings

The images that were acquired using the INCELL2000 were 2048 × 2048 pixels in size at 100 DPI, 16 bit in bit depth and were acquired at 37 °C in an environmental chamber in DMEM (See starvation conditions section). Manufacturer’s settings were used for the excitation and emission. The images that were acquired using the LSM710 had a scaling of ~0.076 µm (i.e 1 pixel = 0.076 µm), 8 bit in bit depth and were acquired at 37 °C in an environmental chamber in DMEM (See starvation conditions section). The excitation wavelengths used: 405, 488, 543 and 639 nm. We used GaAS detectors for detection of the green and red signal using 525/50 and 594/46 filter sets. Blue emission was detected between 410—500 nm and far-red emission was detected between 644–797 nm.

### Image processing and filopodia length measurements

All of the images that were acquired by the dedicated instrument’s software were processed in FIJI. Images were adjusted for brightness and smoothed. Filopodia length was measured manually in FIJI using the “freehand line”.

### Expression and Purification of Myo19 constructs

Expression of constructs were performed in suspension adapted HEK293SF-3F6^70^ (ATCC) cells grown in serum free media EX-CELL (Sigma-Aldrich) or in house made proprietary cell culture media. Myo19^W140V^-3IQ mutant was transiently co-transfected with calmodulin (ratio used 2:1). The transfected cells (initial concentration of 1–2 × 10^6^ cells/ml) were allowed to grow for 2–3 days at 37 °C while shaking (reaching 2–4 × 10^6^ cells/ml) and were then pelleted at 350 × g for 10 min. All protein purification steps were carried out on 4 °C. On the day of purification cell pellets were re-suspended in HaloTag system compatible lysis buffer (20 mM MOPS, 130 mM KCl, 5 mM MgCl_2_, 2 mM EGTA, 2 mM ATP, 0.5 mM DTT, 5% Glycerol, 0.05% NP-40, 2 µM calmodulin, 1 X Promega protease inhibitor cocktail, pH 7.5) to achieve cell density ≈40 × 10^6^ cells/ml and lysed by homogenization in a Dounce homogenizer with a glass pestle by ≈60 strokes. Lysates were supplemented with additional 1 mM MgATP and cleared by ultra-centrifugation at 100,000 × g for 1 h (4 °C). From this point on, the purification followed HaloTag purification system manuals and protocols provided by Promega. Final elutions were concentrated using Millipore (Merck, Darmstadt, Germany) 3 kDa amicon ultra centrifugal filter unit and dialyzed into storage buffer (20 mM MOPS, 75 mM KCl, 5 mM MgCl_2_, 2 mM EGTA, 0.5 mM DTT, 50% Glycerol, pH 7.5). The purified constructs were stored at −20 °C. Myo19-3IQ and Myo19-3IQ^W140V^ concentration were determined using predicted extinction coefficient at 280 nm (λ_ex.coff, WT_ = 105,770 M^−1^ cm^−1^, λ_ex.coff, W140V_ = 100207 M^−1^ cm^−1^, ExPASy ProtParam). The fraction of [active heads] was obtained as for the WT assuming similar efficiency of the expression and purification system to produce WT and mutant constructs (Usaj and Henn, parallel submission).

### Expression and Purification of other proteins

Actin was purified from rabbit or chicken skeletal muscle labeled and gel-filtered over Sephacryl S-300 HR^71^. Ca^2+^-actin monomers were converted to Mg^2+^- actin monomers with 0.2 mM EGTA and 40 µM MgCl_2_ (excess over [actin]) immediately prior to polymerization by dialysis against KMg50 buffer (20 mM MOPS, 50 mM KCl, 2 mM MgCl_2_, 0.2 mM EGTA, 1 mM DTT, pH 7.3 at 25 °C). Phalloidin (1:1 molar ratio) was used to stabilize actin filaments. 7-Diethylamino-3-((((2-maleimidyl) ethyl)amino)carbonyl)coumarin-labeled phosphate-binding protein (P_*i*_BP) was expressed, purified, and labeled as described^72^. Calmodulin was expressed and purified in bacteria as described^73^.

### Steady-state ATPase activity

Steady-state ATPase Activity - The actin-activated steady-state ATPase activity of Myo19-3IQ^W140V^ was measured at 25 ± 0.1 °C in KMg50 buffer supplemented with 2 mM MgATP using the NADH coupled assay^46^ by monitoring changes in absorption at 340 nm. The Myo19-3IQ^W140V^ concentration was 10–50 nM but constant during each individual experiment.

### Stopped-flow Measurements

All experiments were performed in KMg50 buffer with the Hi-Tech Scientific SF-61DX2 stopped-flow apparatus (TgK Scientific Limited, Bradford-on-Avon, UK) at 25 ± 0.1 °C. The concentrations stated through all the text are always final concentrations after mixing (i.e. in the observation cell) unless noted otherwise. Light scattering was measured at 90 ° with excitation at 313 nm. Most time courses shown are of individual, 2000-point transients collected with the instrument in oversampling mode, where the intrinsic time constant for data acquisition is ≈64 µs. Typically, multiple (3 to 5) time courses were averaged before analysis. Time courses displayed fast and slow phases were collected on a logarithmic or split time scale. Using Kinetic Studio software provided with the instrument or with Origin (OriginLab Corporation, Northampton, Massachusetts, USA), time courses of signal (fluorescence, light scattering) change were fitted to a sum of exponentials (Equation 2),

2 $$F(t)={F}_{\infty }+{\sum }_{i=1}^{n}{A}_{i}{e}^{-{k}_{i}t}$$

where *F(t)* is the signal at time *t*, F_∞_ is the final signal value, *A*_*i*_ is the amplitude, *k*_*i*_ is the observed rate constant characterizing the i-th relaxation process, and *n* is the total number of observed relaxations. The value of n was either one (single exponential) or two (double exponential). For mant-nucleotides binding photobleaching was not affecting time courses inside fitting windows (<5 s). The dead time of the instrument determined from the reduction of 2,6-dichlorophenolindophenol with ascorbic acid in absorbance mode was 1 ms. Fitting was limited to data beyond 1 ms to account for the instrument dead time and to exclude data acquired during the continuous flow phase of mixing, as recommended by the manufacturer.

Uncertainties are reported as standard errors in the fits unless stated otherwise and were propagated using the general formula (Equation 3),

3 $$da=\sqrt{{(\frac{\partial a}{\partial {x}_{1}}d{x}_{1})}^{2}+\ldots +{(\frac{\partial a}{\partial {x}_{n}}d{x}_{n})}^{2}}$$

where the experimental measurements x_1_, x_2_… x_n_ have uncertainties dx_1_, dx_2_… dx_n_ and a is a function of x_1_, x_2_… x_n_.

### Nucleotide Binding Kinetics

Time courses of nucleotide binding were acquired under pseudo first-order conditions with [nucleotide] >> [myosin or actomyosin]. Actomyosin samples were prepared by mixing equal molar amount of Myo19-3IQ with actin filaments or, where specified, with [actin]» [myosin]. Myosin and actomyosin samples were treated with apyrase (0.01 unit/ml) and equilibrated on ice for 10 min before measurements. The final apyrase concentration after mixing was 0.005 unit/ml used to deplete ATP and ADP from Myo19-3IQ and Acto∙Myo19-3IQ^46^.

### Kinetic simulations and modeling

Kinetic simulations and modeling of reaction time courses were performed using KinTek Explorer^74, 75^. Simulations of the steady-state reaction time courses were performed according rate constants in Table 1. For the mutant, all the rate constants parameters that were measured were incorporated into the model. *K*′_−AP*i*,_ was the only free parameter that was allowed to be determined by the simulated model until the curve converge to the observed [Actin]-dependence steady-state ATPase. To plot the duty ratio as a function of actin, the fraction of bound and unbound myosin states to actin were directly summed based on the whole reaction simulation mechanism.

### Statistical analysis

Statistical significance between mock and treated groups was performed using two tailed Student’s T-test. We assumed equal variance since the samples originated from the same source. Statistical significance was scored as P ≤ 0.05 *P ≤ 0.01 **P ≤ 0.001***. All presented error bars indicate standard deviation.

### Data availability

No datasets were generated or analysed during the current study.

## Electronic supplementary material

Supplementary Information

Movie S1

Movie S2

Movie S3
