# Supplementary material for: ROS induced distribution of mitochondria to filopodia by Myo19 depends on a class specific tryptophan in the motor domain
Source: Sci Rep. 2017 Sep 14;7:11577. doi: 10.1038/s41598-017-11002-9 (PMC5599611; doi:10.1038/s41598-017-11002-9)
Supplement: Supplementary file 1 — Supplementary Information [file 41598_2017_11002_MOESM1_ESM.doc]

**Supplementary Information**

**ROS induced distribution of mitochondria to filopodia by Myo19 depends on a class specific tryptophan in the motor domain**

Boris I. Shneyer, Marko Ušaj, Naama Wiesel-Motiuk, Ronit Regev, and Arnon Henn*

Faculty of Biology, Technion - Israel Institute of Technology, Haifa, 3200003, Israel

*Address correspondence to: Arnon Henn, Faculty of Biology, Technion - Israel Institute of Technology, Haifa, 3200003, Israel Tel. +947 (4) 8294839; Fax. +947 (4) 8295424; email: arnon.henn@technion.ac.il

**Supplementary Figures**


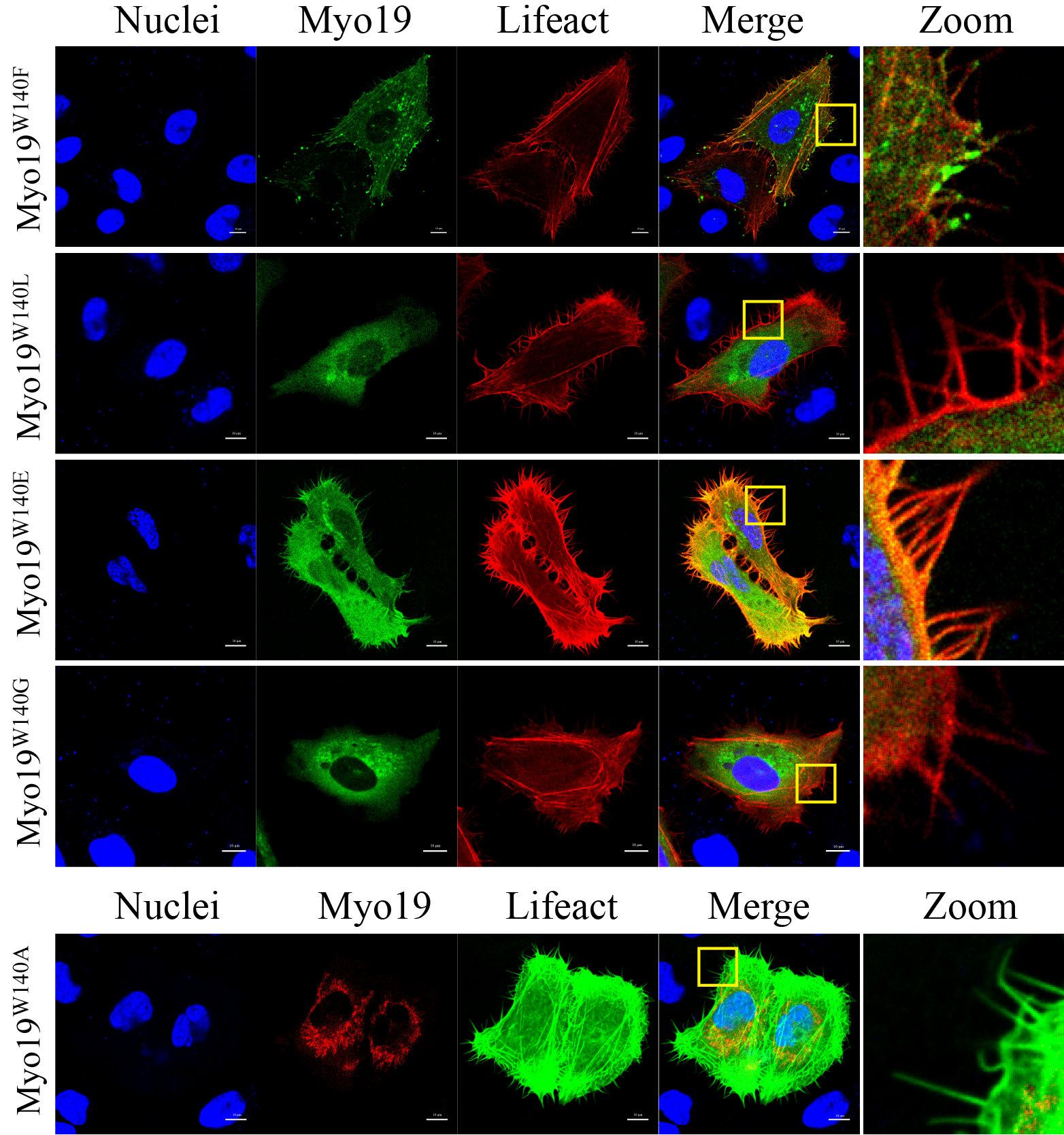
**Figure S1**

| Amino acid at position 140 of *hs*Myo19 | Residue volume (Å^3^) | H_2_O_2_ induced filopodia localization |
| --- | --- | --- |
| W (WT) | 231.7 | + |
| F | 193.5 | + |
| L | 164.6 | - |
| E | 140.8 | - |
| V | 139.1 | - |
| A | 90.1 | - |
| G | 63.8 | - |

**Figure S1: Localization of the different Myo19 mutants in the unique W140 in response to ROS.** U2OS cells were co-transfected with the Myo19 mutants tagged either with emerald or halo, and lifeact tagged either with emerald or ruby to visualize actin and filopodia. The cells were then induced with H_2_O_2_ for two hours to test whether the mutants exhibit the WT filopodial phenotype. Only Myo19^W140F^ maintained the WT phenotype, suggesting that a bulky residue is required at that position. The indicated residue volumes have been adapted from Counterman *et. al*[^1^](#_ENREF_1)


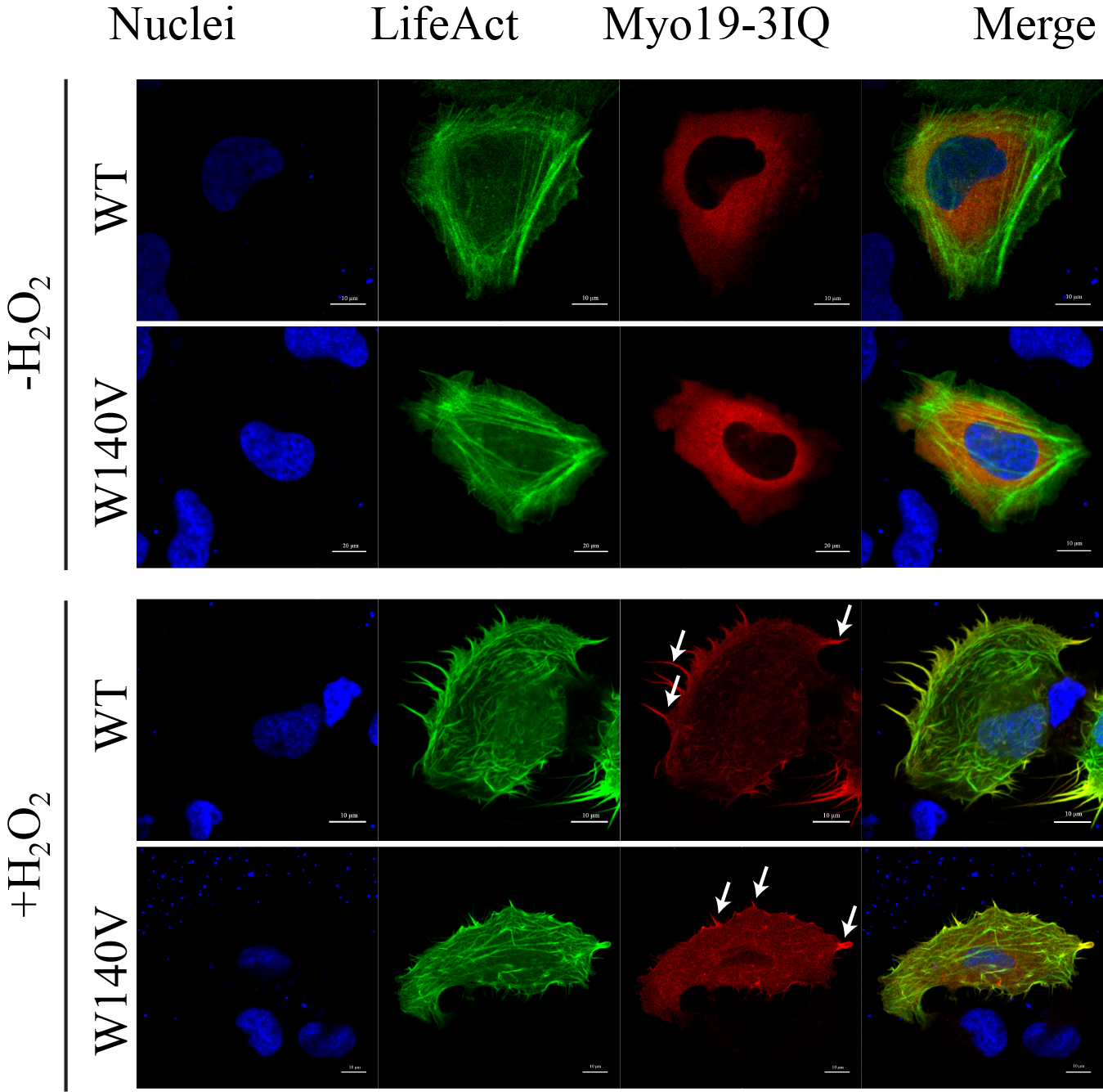
**Figure S2**

**Fig S2: A cargo free Myo19W140V is able to reach filopodia tips.** U2OS cells were co-transfected with emLifeact and either Myo19-3IQ-Halo or Myo19^W140V^-3IQ-Halo constructs and stimulated with H_2_O_2_ to induce filopodia formation. Upper panel top and lower rows are the distribution of the motor constructs under no induction of H_2_O_2_ both WT and mutants are being distributed without localizing to the mitochondria. Unlike the full-length constructs, Myo19-3IQ decorated the entire filopodia length (bottom panel, top row, marked with white arrow). Similarly, Myo19^W140V^-3IQ was able to reach filopodia in a similar fashion, indicating that it possesses motor activity. This is in contrast to the lack of localization of the full-length Myo19^W140V^ inability to power mitochondria movement due to its altered mechanochemical cycle and. Blue – nuclei, green – lifeact, red – Myo19-3IQ-Halo or Myo19^W140V^-3IQ-Halo, white arrows – filopodia positive for Myo19. Bar is 10 µm.

**Figure S3**

**
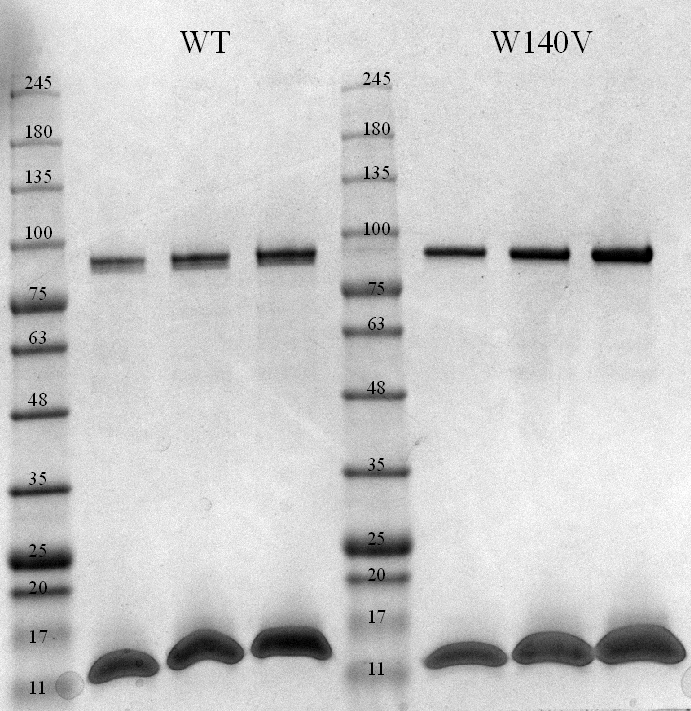
**

**Figure S3:** **Coomassie Blue stained SDS-polyacrylamide gel of the purified constructs.** Myosins heavy chain (97.3 kDa) (upper bands) and the associated calmodulin light chain (16.3 kDa) (lower bands) for Myosin 19-3IQ (WT) and Myo19-3IQ^W140V^ (W140V) for three different gel loading.

F**igure S4**

**
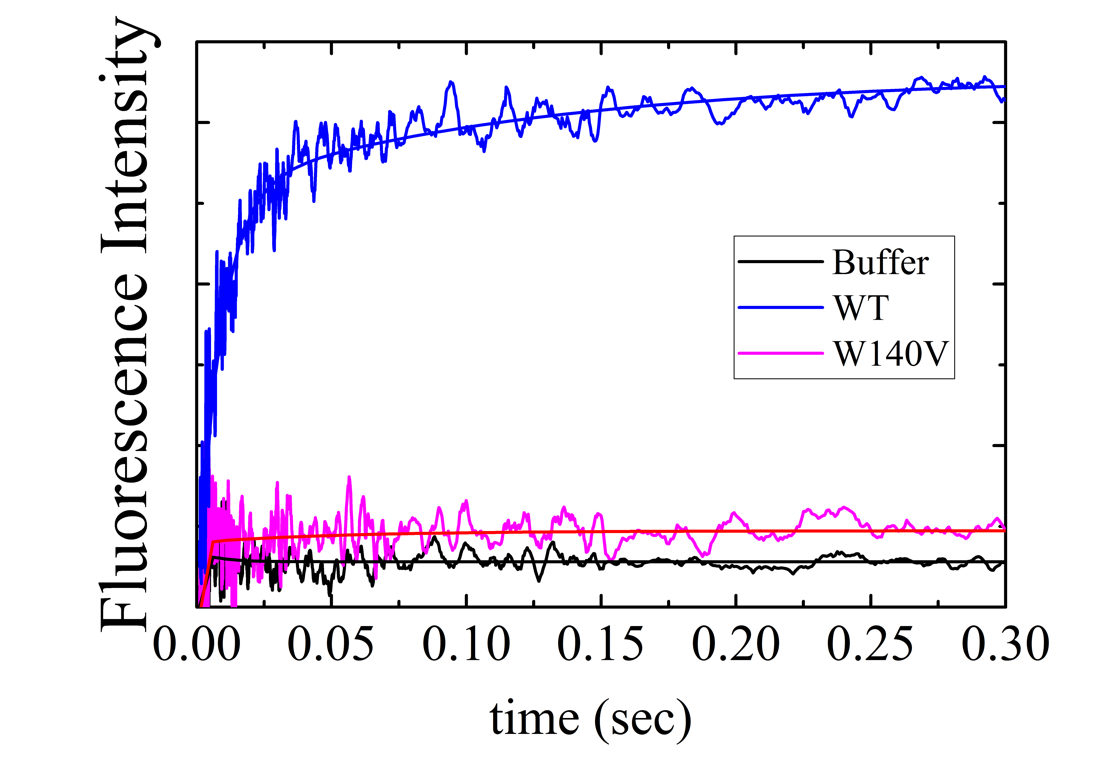
**

**Figure S4: mantADP binding kinetics to Myo19-3IQ and Myo19-3IQ^W140V^.** Time courses of 2’/3’-mantADP fluorescence change after mixing 200 nM Myo19-3IQ (WT) or Myo19^W140V^-3IQ with the assay buffer (black line) or with 10 µM 2’/3’-mantADP (blue and purple line). No fluorescence increase was detected with the mutant Myo19-3IQ^W140V^ upon mantADP binding.

**Figure S5**

**a) b)**

**c) d)**

**Figure S5**: **Residual analysis for single and double exponential fitting for ATP induced dissociation of Acto·Myo19-3IQ^W140V^ by light scattering.** The residual analysis of the fitting to single (black scattered plot) or double (blue scattered plot) exponential functions of the transients obtained upon mixing Acto·Myo19-3IQ**^W140V^** (0.25 µM) with 1.95 (**a**), 3.9 (**b**), 7.8 (**c**) and 31.25 (**d**) µM ATP final concentration (the experiments are fully described in Fig. 7).

**Movie S1: Time-lapse imaging of U2OS cells expressing emMyo19 and Ruby-Lifeact.** Time lapse imaging was used to follow emMyo19 localization to filopodia tips. U2OS cells were transfected with emMyo19 and Ruby-Lifeact to visualize Myo19 and actin, respectively. The cells were stimulated with H_2_O_2_ and imaged for 1.5 hours every ~30 seconds using confocal microscopy (Scale bar, 20 μm).

**Movie S2: ‘Catch up’ type movements of emMyo19 within filopodia.** Zoom-in to the cells in movie S1 showing movement of emMyo19 from interior actin cortex towards the filopodia shaft. Note the bottom filopodia where Myo19 reaches the filopodia base and stalls where filopodia continues to grow (Scale bar, 10 μm).

**Movie S3: Forward movement followed by rearward movement of Myo19 on retracting filopodia.** Zoom in to the parts of the cell in S1 showing a retracting filopodium. Myo19 following back into the cell body prior to filopodia retraction (Scale bar, 10 μm).

**Table S1:** DNA oligos used for the site-directed mutagenesis to prepare the full-length Myo19^W140X^ and the Myo19^W140X^-3IQ constructs.

| Construct | Oligos (5’- 3’) |
| --- | --- |
| Myo19^W140F^ | CACGTCTCGCTGCCTAATGAAGTTCT |
|  | CATGTCTTTCCAGCACCACTCTCTCC |
| Myo19^W140L^ | AACGTCTCGCTGCCTAATGAAGTT |
|  | AATGTCTTTCCAGCACCACTCTCTC |
| Myo19^W140E^ | AGACGTCTCGCTGCCTAATGAAG |
|  | CTGTCTTTCCAGCACCACTCTCTC |
| Myo19^W140V^ | TGACGTCTCGCTGCCTAAT |
|  | CTGTCTTTCCAGCACCACTCT |
| Myo19^W140A^ | CGACGTCTCGCTGCCTAAT |
|  | CTGTCTTTCCAGCACCACTCTC |
| Myo19^W140G^ | GGACGTCTCGCTGCCTAATG |
|  | CTGTCTTTCCAGCACCACTCTCTC |

**Supplementary References**

1. Counterman, A. E. & Clemmer, D. E. Volumes of Individual Amino Acid Residues in Gas-Phase Peptide Ions*. Journal of the American Chemical Socie*t**y 1**21, 4031-4039, doi:10.1021/ja984344p (1999).
